# Identifying barriers to early presentation in patients with locally advanced breast cancer (LABC) in Northern Singapore: Qualitative study

**DOI:** 10.1371/journal.pone.0252008

**Published:** 2021-05-25

**Authors:** Ding Yi Ng, Lorraine Tudor Car, Marcus Jia Ming Ng, Junde Lu, Joelle Leung, Tiong Thye Goo, Clement Luck Khng Chia

**Affiliations:** 1 Lee Kong Chian School of Medicine, Nanyang Technological University, Singapore, Singapore; 2 Department of Primary Care and Public Health, School of Public Health, Imperial College London, London, United Kingdom; 3 Yong Loo Lin School of Medicine, National University of Singapore, Singapore, Singapore; 4 Department of General Surgery, Khoo Teck Puat Hospital, Singapore, Singapore; Medical University Innsbruck, AUSTRIA

## Abstract

**Introduction:**

Breast cancer is the leading cause of death in Singaporean women, with advanced stage rendering a poorer prognosis. This study aims to explore the barriers to early presentation, information needs and sources in patients with locally advanced breast cancer (LABC).

**Materials & methods:**

A convenience sample of patients who presented with locally advanced breast cancer to the Department of General Surgery in a teaching tertiary hospital were recruited for the study. We conducted semi-structured interviews face to face with the recruited patients. We recorded the interviews, transcribed them verbatim and analysed using thematic content analysis.

**Results:**

Twenty-three participants were recruited of which 12 were Chinese and 11 were Malay women. Mean age was 60 years (± 13 SD). The most common knowledge barrier resulting in delay was the misconception that a breast lump must be painful to be malignant. Other knowledge barriers include the lack of knowledge and misinformation from the internet or other social media platforms. Some perceived barriers include fear of diagnosis, fear of treatment and fear of imposing financial burden on family members. A significant proportion of participants were also not aware of a national breast screening programme.

**Conclusions:**

Our study has found that barriers to early presentation of women with locally advanced breast cancer remain similar and have persisted over the years despite targeted efforts. There is a need for a rethink of existing strategies and to develop new innovative ways to reach out to this group of patients.

## Introduction

Breast cancer is the leading cause of cancer death in women in Singapore for the past five decades [1]. Since 1968, there has been a steady rise in the age-standardised incidence rate for invasive breast cancer (IBC) from 20.1 per 100,000 population in 1968–1972 to 69.8 per 100,000 population in 2013–2017 [1]. An increasing trend of stage four IBC from 9.8% in 2008 to 11.6% in 2017 [1] is also worrisome.

This is despite having an established breast screening programme in Singapore (BreastScreen Singapore) implemented in 2002. Women above the age of 40 are invited for mammogram screenings yearly between the age of 40 to 49 years old and every two years for patients aged 50 to 69 years old to increase pre-cancerous detection [2] which has been associated with 24% reduction in mortality [2]. However, the National Health Survey done in 2010 revealed that the breast cancer screening rates in Singapore is less than ideal with only 40.4% of women aged 50–59 and 38.1% of women aged 60–69 having had a mammogram done in the past 2 years [3]. Overall, only 66.3% of women aged 50–69 had ever gone for a mammogram despite 90.9% of the population [3] surveyed were aware of the utility of the mammogram, indicating the need to further understand the reasons for the low uptake rates.

A delay in presentation and treatment is costly for the patient with a recent study by Ho et al. [4] indicating that delayed treatment of more than 90 days post diagnosis has been associated with poorer survival outcomes compared to patients who received treatment within 30 days post-diagnosis. Researchers have attempted to shed light behind the reasons for delayed presentation and explore strategies to tackle this problem [5, 6] with earlier studies finding that delayed presentation often arise from misconceptions about the disease pertaining to screening and treatment. A study by Lim et al., [7] that explored the reasons for late presentation in women with breast cancer from both Singapore and Malaysia found poor symptom interpretation, fear of diagnosis, misinformation from online resources and preference for alternative and traditional medicine to be the main reasons.

Over the years, policy makers and healthcare institutions have developed coordinated efforts to address some of these issues. Khoo Teck Puat Hospital is a 761-bed general and acute care hospital serving the North and heartlands of Singapore. Data published by the Singapore Census of Population 2010 [7] indicated that 45.67% of the population in the North (Yishun, Sembawang and Woodlands zones) have at least post-secondary education qualifications. In Singapore, the month of October has been stipulated Breast Cancer Awareness month and breast cancer public forums are organised at various locations in the country to raise awareness and educate the public on the local incidence and pathophysiology of disease, screening methods and treatment options. Mammobus Singapore was also recently introduced to bring screening to the heartlands with the aim of increasing accessibility to these screenings.

As such, with this slew of targeted efforts aimed at encouraging screening and early presentation, the aim of this study is to explore the barriers, information needs and sources of patients with LABC to determine the underlying reasons as to why these issues persists.

## Methods

### Study design and participant recruitment

A qualitative study consisting of semi structured interviews was used to explore barriers to earlier presentation in women with locally advanced breast cancer in Singapore.

This study has attained ethics approval from the National Healthcare Group Domain Specific Review Board (DSRB).

Patients with locally advanced breast cancer (LABC) at first presentation were recruited from June 2018 to January 2020 for the study in the Department of General Surgery in a teaching tertiary hospital, Khoo Teck Puat Hospital. LABC is defined as any T3 or T4 breast tumour or involvement of axillary lymph nodes confirmed on pathology [8]. The patients recruited were females of any age and ethnicity.

We employed convenience sampling. Interested patients were provided with information about the study before written and informed consent was obtained.

### Interview process

A qualitative one-to-one interview was undertaken to explore the rationales for late presentation that could have been influenced by patients’ knowledge and perception of the disease.

The one-to-one interviews were conducted using an open-ended semi-structured questioning style that was guided by an interview guide (S1 Appendix) which allowed for open dialogue between the stakeholders [9].

Transcription of the recording was subsequently done and the coding process of the interviews was initiated.

Burnard’s thematic content analysis [10] was employed and was conducted in multiple stages. First, open coding was done for the transcripts, labelling pertinent phrases or words with a theme. Subsequently, similar themes were grouped together and duplicates removed.

We continued the interviews until no new themes and experiences emerged from the collected data. After 21 participants were interviewed, there were no new emergent themes. Data saturation [11] was deemed to have been met after 23 participants were interviewed in total.

## Results

A total of 23 patients were recruited and their interviews were transcribed and coded. The demographic breakdown for these 23 participants is represented in Table 1.

**Table 1:** Demographics of participants.

Categories	Frequency
	Sample size n = 23
Ethnicity	
Chinese	12
Malay	11
Mean Age (years ± SD)	60 ± 13
Marital status	
Single	5
Married	18
Stage of tumor	
Two	3
Three	10
Four	10

The study looked into the current sources of health-related information that patients subscribe to, with some patients indicating more than one source of information. Fifteen participants cited online resources as one of their information sources, nine participants cited media (radio, television and print), six participants cited general practitioners, five participants cited friends, five participants cited relatives and spouses and lastly, one cited colleagues.

Reasons for not participating in breast screening programmes (BreastScreen) were also elicited. The predominant reasons for participants not attending the BreastScreen programmes were either unawareness of the existence of Government-subsidised programmes (n = 6) or fear of pain that mammogram induces (n = 7). Other reasons included neglect (n = 5), the lack of belief in mortality benefit of mammograms (n = 2), long waiting times (n = 1), fear of radiation exposure (n = 1) and those not within age range of the screenings (n = 1).

Finally, the impetus for eventual presentation to healthcare facilities were explored with the major contributory factor being that of family persuasion. Other reasons include sudden onset of pain or development of skin changes.

### Pertinent themes

The transcripts of each interview were coded and each theme for barriers to early presentation is presented below with their relevant quotes and the frequency for each theme.

**Fig 1:**
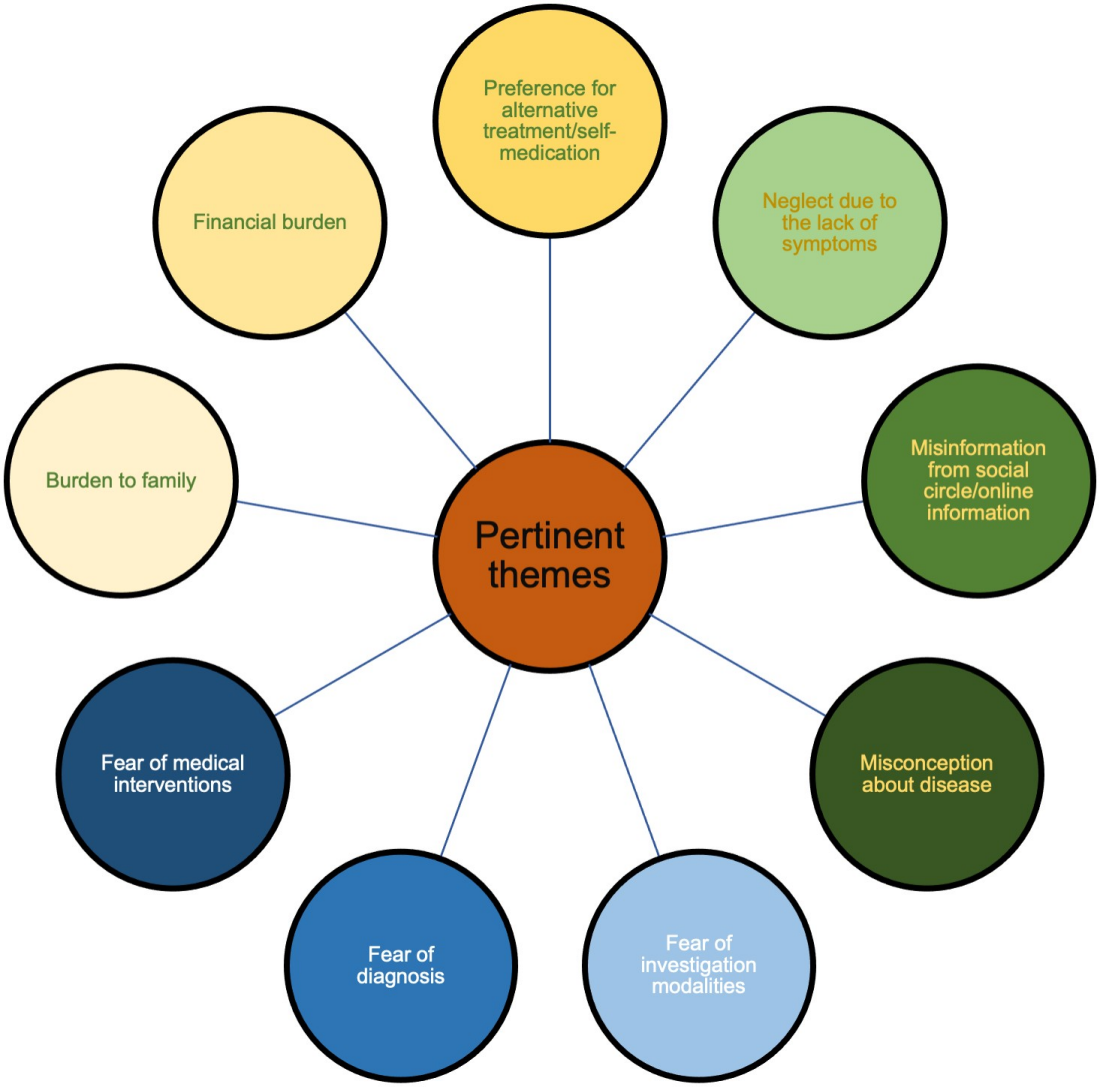
Pertinent themes elucidated from the patients.

The themes were grouped into three broader barriers to presentation namely, 1) knowledge, 2) perception and fear and 3) financial and social reasons. This is represented in Fig 1.

#### Knowledge

*Neglect due to lack of symptoms*. Seventeen women (74%), eight Malay and nine Chinese mentioned that the neglect of the palpable breast lump due to the lack of pain and other symptoms such as palpable lymph nodes, growth of the mass or skin changes.

“I only came because my breast started to get red and painful. It also had some pus.”

Patient 3, Chinese

“Before the size growth (of the breast) there was actually a lump. So (only) when (my breast grew) bigger on one side, then (I decided to go to doctor)”

Patient 11, Malay

*Preference for alternative treatment/self-medication*. Nine women (39%), five Malay and four Chinese, either sought for alternative medications that they knew about through their social circle or via social media, or believed in other therapies such as changing their diet or warm compress.

"After the lump appeared, I went to the Chinese herbal stores to buy medications in hope that the lump would become smaller."

Patient 3, Chinese

"I used a hot towel to compress (the) lump"

Patient 22, Malay

*Misinformation from social circle/online information*. Eight women (35%), three Malay and five Chinese, mentioned that they acquire their information about IBC either through their friends or from online sources. With one woman mentioning that she ignored the breast lump as it did not fit the symptoms as stated online.

“My friend who did an operation to remove her breast repeatedly told her not to remove and do any operation”

Patient 2, Chinese

“Then after that I looked through the internet, then the symptoms in terms of cancer, or that. I didn’t find it that my lump can become cancerous”

Patient 5, Malay

*Misconception about the disease*. Seven women (21%), three Malay and four Chinese, mentioned misconceptions they had about IBC that were derived from either their health beliefs or lack of knowledge about the disease. One of them thought it was normal to have breast lump after menopause.

"Yes, I think it can be prevented by reducing our stress levels as I just had menopause recently and I thought it would be normal to have a lump"

Patient 3, Chinese

"Didn’t know there will be a lump.”

Patient 20, Malay

#### Perception and fear

*Fear of investigation modalities*. Ten women (43%), five Malay and five Chinese, have mentioned that they are afraid of the pain of mammogram screenings that they have either experienced first-hand or through hear-say from their social circles. One of the patient also mentioned that she was afraid that the mammogram may cause seeding of malignant cells.

"I mean they will be pressing the breast, or whatever. That’s why I thought it’s painful, or what, that’s why I didn’t go for it. Also there is some (sources), they write that they will be pressing the (breast lump), then the cancer cells will travel (everywhere)"

Patient 5, Malay

“(My friend) say mammogram is very painful!”

Patient 7, Chinese

*Fear of medical intervention*. Twelve women (52%), six Malay and six Chinese, have mentioned that they are daunted by the treatment that they might receive either because of the experiences of their friends and relatives or personal preconceived notions of treatments.

"Scared because her friend removed a lump and passed away”

Patient 1, Chinese

“(I am most scared of) the side effect and the uncertainty (of the chemotherapy)"

Patient 11, Malay

*Fear of diagnosis*. Eight women (35%), three Malay and five Chinese, mentioned that they were afraid of their confirmed diagnosis which could they either viewed as incurable or was afraid of burdening their family members with the new diagnosis

“I’m afraid to see the doctor. Because I’m afraid if they say I’ve got cancer“

Patient 5, Malay

“Scared (of going to the doctor). Because sometimes we get negative answers.”

Patient 8, Chinese

#### Social and financial

*Burden to the family*. Six women (26%), three Malay and three Chinese, mentioned about being too afraid of being dependent on their families and hence many ignored the symptom hoping not to alarm their families. Some of them also had ongoing family issues such as a sick family member or were in poor financial situation.

“Because I was really scared of disturbing everyone, so I just tried to ignore”.

Patient 6, Malay

“It’s just I feel like I do not want to add my burdens to other people”

Patient 14, Chinese

*Preference for female doctor*. Five women (22%), three Malay and two Chinese, mentioned their preference for female doctors.

“Sometimes it’s when you see the male doctor also, if he is male doctor also. That is also one that block me (from opening up)”

Patient 8, Chinese

“Because maybe he’s a male doctor. I feel a bit shy to ask certain questions.”

Patient 14, Chinese

*Financial burden*. Four women (17%), two Malay and two Chinese, mentioned difficult financial situations in their family that would be exacerbated with their diagnosis and subsequent treatments. This impeded them from seeking treatment.

“I am the sole breadwinner of the family”

Patient 15, Malay

“Then who is going to pay for me if I am going to stay in the hospital?”

Patient 14, Chinese

## Discussion

Locally advanced breast cancer (LABC) is a significant healthcare problem in Singapore and is associated with a worse prognosis of a 15% reduction in five-year survival rates as compared to early breast cancers [12]. This is the first local study in Singapore as far as the authors are aware on patients with LABC to investigate barriers to delayed presentation, information needs and sources in this group of patients. Our results revealed that barriers in delayed presentation can be divided into three main broad themes of 1) knowledge deficit, 2) fear and perception of diagnosis, investigations and treatment, 3) financial and social factors. These factors are often closely intertwined with a knowledge deficit propagating fear and perception of the disease and false assumptions of the financial and social impact that the diseases would have on the patient and her family.

The fear of diagnosis of cancer and treatment were similarly found in the study by Chang et al. [13], where 35% of Singaporeans thought cancer was uniformly fatal and many were also afraid of mastectomy and side effects associated with chemo-radiotherapy. This resulted in delayed presentation and the pursuit of alternative therapies such as Traditional Chinese Medicine which further exacerbates the delay. More importantly, many of these fears were propelled by misconceptions that arose from online sources and social circles who lack accurate knowledge regarding the latest progress in breast cancer treatment and reduction in systemic toxicity and side effects related to chemo-radiotherapy. We believe that public education outreach efforts that relay accurate and updated information on breast cancer and its treatment will help to clarify and debunk any myths thereby allaying fear surrounding diagnosis and treatment.

In addition, we also found a quarter of patients in this study were not aware of the presence of a national breast cancer screening programme. Common misconceptions about investigations include the risk of seeding of malignant cells associated with biopsy and failure to understand the need for further follow-up even if the first screening is normal.

This highlights the importance of enhanced awareness campaigns for breast screening programmes. It is also imperative to stress the importance of regular follow-up screening in women with an initial normal mammogram result. Interestingly, incentives for breast screening had been studied previously and found to have a positive impact on encouraging second screening visits [14].

In the current study, some of the erroneous beliefs stem from unreliable online sources that are not peer reviewed. Participants report social media platforms that publicise non-scientific alternative medicine with claims of being less invasive and lower in cost that resulted in delay in presentation to the hospital. This is consistent with Lim et al. study [15] that found unreliable online resources being a major contributory factor for misinformation. Another source of misinformation arise from social circles of the patient. Family and friends have contributed to delayed presentation when they wrongly reassured patients based on their own experiences or discourage treatment based on their own perception. On the contrary, positive encouragement from close social circles could increase attendance for breast screening programmes. We believe that a more collective and family centric approach should be pursued in terms of education which has also been shown to be effective in diabetes management [16]. Through collective enforcement of health-seeking behaviour, increasing awareness of screening and knowledge of IBC, these will prevent propagation of misinformation among social circles.

Credible, free online resources such as government hospital websites [17–19], the Singapore Cancer Society [20] or Singapore Breast Cancer Foundation [21] are already available to the Singapore public. To improve their reach and engagement of the public, the websites content could be made more interactive or be presented in different languages that is applicable to multi-ethnic Singapore. The use of interactive media has been shown to be effective in improving knowledge and shared decision making in end-stage renal failure patients [22] and could be replicated in breast cancer.

The fear of reliance and imposing a financial burden on other family members have also been elucidated in previous studies [23]. This is despite many government funded financial assistance schemes available for the needy such as Medisave, a national medical savings scheme which covers up to SGD 1,200 per outpatient chemotherapy cycle and up to SGD 2,150 for a mastectomy [24]. Other avenues of medical coverage include Medishield Life [25], a universal medical insurance scheme which helps to cover up to SGD 3,000 per chemotherapy cycle and when applicable, Medifund [26] which is an assistance scheme for lower-income families could also alleviate financial concerns. We suggest that these information be tagged to either media, radio broadcasts, newspapers, magazines or government websites promoting breast cancer screening as relevant and pertinent information that will influence patients’ decisions to come forward.

The strength of the study lies in the in-depth interview approach and open-ended style of questioning by interviewers that encourage more forthcoming responses. Although this study is conducted within the social cultural context in Singapore, it is interesting that our findings are similar to a United Kingdom (UK) study [27] pertaining to knowledge barriers and false perceptions and fear. The difference is that Singapore patients have a more pronounced fear of being a financial and social burden which could perhaps be explained by the difference in healthcare subsidy models of the UK National Health Service and locally. In comparison to Malaysia, a neighbouring country to Singapore, findings for reasons for delayed presentation were similar with the exception of the fear of misdiagnosis by healthcare professionals in Malaysia [15].

A limitation of this study is that we did not include socioeconomic background of participants such as educational level or income level. Poor health literacy have been found to be associated with educational levels [7] and may be an important contributory factor that should be evaluated in future studies. A second limitation of the study is the use of convenience sampling, reducing the generalisability of the results from this study. Another limitation would be the absence of Indian minority ethnic group being represented in this study. In fact, the ethnic distribution of LABC patients in this study (47.8% Malay and 52.2% Chinese) is disproportionate to the ethnic distribution of Singaporean residents (13.2% Malay and 74.8% Chinese) [28]. A previous study investigating the outcomes of breast cancer in Malay women suggested that there is a greater predisposition for Malay women to present with LABC [29]. We acknowledge that this may be limited by the small sample size and geographic location where the hospital is situated but this is a phenomenon that should be further examined in future studies.

In conclusion, we found that barriers to early presentation in women with LABC are similar to those identified in previous studies and have persisted over the years. Misinformation through unreliable online sources and social circles further compound the problem. There is a need for evaluation of and implementation of new strategies to engage women with LABC and encourage earlier presentation.

## Supporting information

S1 Appendix Study interview guide and questionnaire. (DOCX)

S1 Dataset (DOCX)

## References

[1] Singapore Cancer Registry (50 year anniversary monograph). 2017;

[2] Ministry of Health Practice Guidelines—Cancer Screening. Ministry of Health, Singapore. 2010. 20358158

[3] Epidemiology & Disease Control Division, Ministry of Healthy S. National Health Survery. 2010;

[4] Ho PJ, Cook AR, Binte Mohamed Ri NK, Liu J, Li J, Hartman M. Impact of delayed treatment in women diagnosed with breast cancer: A population-based study. Cancer Med. 2020;9(7):2435–44. 10.1002/cam4.2830 32053293 PMC7131859

[5] Kerr-Cresswell DM, Fitzgerald B, Fergus K, Gould J, Lenis M, Clemons M. Why so late? Presentation delay in locally advanced breast cancer (LABC). J Clin Oncol. 2005;

[6] Malhotra C, Bilger M, Liu J, Finkelstein E. Barriers to Breast and Cervical Cancer Screening in Singapore: a Mixed Methods Analysis. Asian Pac J Cancer Prev. 2016; 27644635

[7] Department of Statistics, Ministry of Trade and Industry R of S. Singapore Census of Population 2010. 2010;71–2.

[8] NCCN. NCCN Clinical Practice Guidelines in Oncology (NCCN Guidelines^®^) Breast Cancer. Version 2.2015. 2015.

[9] Steiner E, Xu K. Binge-watching motivates change: Uses and gratifications of streaming video viewers challenge traditional TV research. Converg Int J Res into New Media Technol. 2018;

[10] Burnard P, Gill P, Stewart K, Treasure E, Chadwick B. Analysing and presenting qualitative data. Br Dent J. 2008;204(8):429–32. 10.1038/sj.bdj.2008.292 18438371

[11] Fusch PI, Ness LR. Are we there yet? Data saturation in qualitative research. Qual Rep. 2015.

[12] Vondeling GT, Menezes GL, Dvortsin EP, Jansman FGA, Konings IR, Postma MJ, et al. Burden of early, advanced and metastatic breast cancer in The Netherlands. BMC Cancer. 2018; 10.1186/s12885-018-4158-3 29514651 PMC5842550

[13] Chang G, Chan CW, Hartman M. A commentary on delayed presentation of breast cancer in Singapore. Asian Pacific J Cancer Prev. 2011;12(6):1635–9. 22126512

[14] Merrick EL, Hodgkin D, Horgan CM, Lorenz LS, Panas L, Ritter GA, et al. Testing novel patient financial incentives to increase breast cancer screening. Am J Manag Care. 2015; 26633251

[15] Lim JNW, Potrata B, Simonella L, Ng CWQ, Aw TC, Dahlui M, et al. Barriers to early presentation of self-discovered breast cancer in Singapore and Malaysia: A qualitative multicentre study. BMJ Open. 2015;5(12):1–9. 10.1136/bmjopen-2015-009863 PMC4691764 26692558

[16] Teufel-Shone NI, Drummond R, Rawiel U. Developing and adapting a family-based diabetes program at the U.S.-Mexico Border. Prev Chronic Dis. 2005; 15670473 PMC1323323

[17] HealthHub. Breast Cancer Information [Internet]. 2020 [cited 2020 Apr 14]. https://www.healthhub.sg/a-z/diseases-and-conditions/20/breastcancer

[18] Singhealth. The landscape of breast cancer screening and treatment in Singapore–how well do we know it [Internet]. 2016 [cited 2020 Apr 14]. https://www.singhealth.com.sg/news/medical-news/landscape-breast-cancer-screening-treatment-singapore

[19] National Cancer Centre Singapore. Breast Cancer—What it is [Internet]. [cited 2020 Apr 14]. https://www.nccs.com.sg/patient-care/conditions-treatments/breast-cancer/

[20] Singapore Cancer Society. Breast Cancer [Internet]. [cited 2020 Apr 14]. https://www.singaporecancersociety.org.sg/learn-about-cancer/types-of-cancer/breast-cancer.html

[21] Breast Cancer Foundation S. What is Breast Cancer? [Internet]. [cited 2020 Apr 14]. https://www.bcf.org.sg/learn-more/what-is-breast-cancer/

[22] Chiou CP, Chung YC. Effectiveness of multimedia interactive patient education on knowledge, uncertainty and decision-making in patients with end-stage renal disease. J Clin Nurs. 2012; 10.1111/j.1365-2702.2011.03793.x 21883569

[23] Seetoh T, Siew WF, Koh A, Liau WF, Koh GCH, Lee JJM, et al. Overcoming Barriers to Mammography Screening: A Quasi-randomised Pragmatic Trial in a Community-based Primary Care Setting. Ann Acad Med Singapore. 2014; 25588917

[24] Ministry of Health S. Ministry of Health Singapore, Medisave.

[25] National Cancer Centre Singapore. National Cancer Centre Singapore, Medishield Life Payment Option.

[26] Ministry of Health S. Ministry of Health, Medifund.

[27] Jones CEL, Maben J, Lucas G, Davies EA, Jack RH, Ream E. Barriers to early diagnosis of symptomatic breast cancer: A qualitative study of Black African, Black Caribbean and White British women living in the UK. BMJ Open. 2015; 10.1136/bmjopen-2014-006944 25770231 PMC4360845

[28] Data.gov.sg. Singapore Residents By Age Group, Ethnic Group And Gender, End June, Annual [Internet]. [cited 2020 Mar 19]. https://data.gov.sg/dataset/resident-population-by-ethnicity-gender-and-age-group?view_id=8ff89d3f-48c8-46e4-8a4d-a8b9f152976f&resource_id=f9dbfc75-a2dc-42af-9f50-425e4107ae84

[29] Xin WR, Kwok LL, Yong WF. Screening uptake differences are not implicated in poorer breast cancer outcomes among Singaporean Malay women. J Breast Cancer. 2017; 10.4048/jbc.2017.20.2.183 PMC5500402 28690655

