# Supplementary material for: Identifying barriers to early presentation in patients with locally advanced breast cancer (LABC) in Northern Singapore: Qualitative study
Source: PLoS One. 2021 May 25;16(5):e0252008. doi: 10.1371/journal.pone.0252008 (PMC8148318; doi:10.1371/journal.pone.0252008)
Supplement: S1 Appendix — (DOCX) [file pone.0252008.s001.docx]

**Health-related information sources used by the interviewees**

The following questions are designed to understand more about general and breast cancer-focused information sources that patients with locally advanced fungating breast cancer use*.*

1. General sources of health-related information

- When you have questions about your health, treatment or an illness, where do you usually search for answers?
- Do you manage to find answers? If yes, where if no, what do you do then?
- How would you seek answers (if participant does not mention the internet specifically prompt them and ask whether they’d also seek information on the internet)? Do you have any preferred health-information websites that you use?
- When do you turn to your family for help in answering these questions?
- What about your friends?
- Are there any other sources of health-related information do you use?
- Do you feel comfortable asking your doctor questions?
- What are the most helpful sources of information on health for you? Why?

1. Breast cancer-related sources of information

- Did you have any breast cancer-related questions before your diagnosis?
- What kind of questions did you have (e.g. on treatment, symptoms, outcomes etc.)?
- Have you searched for breast cancer-related information?
- Which sources did you use?
- Did you manage to obtain all relevant information? Was this breast cancer-related information useful? If not, why?

**Information needs and preferences**

The following questions are designed to understand breast cancer-related information needs and preferences of patients’ with locally advanced fungating breast cancer.

1. Breast cancer-related information needs are preferences

- What other information would you have liked to receive regarding breast cancer? Is there anything else that you would like to know about breast cancer (e.g. treatment, side effects, prognosis, whether it is inherited)?
- What other information about breast cancer would you like to receive in future? How would you like to receive information (e.g. computer, mobile phone, leaflet, newspaper etc.)?
- How often would you like to receive the information?

**Barriers to early presentation of breast cancer**

The following questions are designed to understand the barriers to early presentation and healthcare seeking in the patients with locally advanced fungating breast cancer.

1. Potential reasons for the delayed presentation due to breast cancer-related misconceptions

- Are you aware of the existing Singapore breast cancer screening programmes?
- What do you think is the purpose of screening programme? Have you taken part in this programme? If not, why?
- Do you think breast cancer can be prevented? If not, why?
- Do you have any family members with breast cancer?
- How did their experience make you feel? Did their illness in any way influence your decisions about seeking help?

1. (Self-)diagnosis and timing to seek for treatment option

- When did you first notice a change in your breasts?
- Could you please tell me more about it (e.g. timing, type of changes)?
- Did you go to the doctor as soon as you noticed the changes in you breast? If not, why?
- How long did you wait to see the doctor?
- Did you notice any other changes in your breasts in the meantime?
- How did they make you feel? Why did you decide to see the doctor in the end?
- What would have made you come and see the doctor earlier than you did?

1. Potential health system-related reasons for delayed presentation

- Where there any problems in booking an appointment with the doctor.
- How long did you have to wait to see the doctor after booking an appointment?
- Were you willing to talk about your breast chances with any doctor.
- Did you go to see you family physician first?
- Did you prefer to see a specific doctor? Do you have any preferences in relation to you doctors (e.g. their age, gender, other characteristic)

1. Potential personal or other reasons for the delayed presentation

- Did you seek for alternative treatment options other than seeing the doctor? For example, traditional Chinese medicine or self-medication?
- Did seeing a doctor put a financial strain on you?
- Did you share any information about your breast changes with your family and friends? If not, why?
- What is your view on the breast cancer treatment?
- Were you in any way afraid of the breast cancer treatment that you may need to undergo?
- Are there any other reasons why you have not come to see the doctor earlier?
