# Supplementary material for: Identifying barriers to early presentation in patients with locally advanced breast cancer (LABC) in Northern Singapore: Qualitative study
Source: PLoS One. 2021 May 25;16(5):e0252008. doi: 10.1371/journal.pone.0252008 (PMC8148318; doi:10.1371/journal.pone.0252008)
Supplement: S1 Dataset — (DOCX) [file pone.0252008.s002.docx]

# S1 Dataset

Pertinent Quotes

Patient 1 (CHINESE)

“never searched for information as i wasnt ill, had no symptoms”

“I don’t go because I’ve heard that the mammogram hurts and I’m afraid of it/ I am afraid (of going to the doctor)”

"Scared because her friend removed a lump and passed away”

“(doesn’t want to do chemo/ treatment as she wants to live a pain-free life)"

“afraid it was breast cancer”

"I feel like I’m bothering the doctor if I asked”

“Never asked anyone about the lump"

Patient 2 (CHINESE)

“Mainly because (she’s) not in pain. She finds that only when she’s in pain she will visit a doctor”

“doesn’t dare to go for the screening because (she’s) scared of pain”

"she thinks that the treatment (her aunt did) made her cancer spread throughout the body”

“she doesn’t dare to tell the doctor because the doctor might insist on an operation ."

“Her friend who did an operation to remove her breast repeatedly told her not to remove and do any operation”

“If there’s no pain, she have no questions or worry”

"Because of a relative who goes for checks regularly, she still passed away after these scans”

“Scared because her friend removed  a lump and passed away"

Patient 3 (CHINESE)

“(I would have seen the doctor) If I had experienced pain”

“The treatment is daunting”

"Yes, I think it can be prevented by reducing our stress levels”

“as I just had menopause recently and I thought it would be normal to have a lump"

Patient 4 (CHINESE)

“I only came because my breast started to get red and painful. It also had some pus.”

"When you see chemo patients, you know they suffer”

“I also had a sister-in-law who had breast cancer. She had surgery and chemotherapy. She seemed to suffer a lot"

"I go for traditional medicine check-ups occasionally

“After the lump appeared, I went to the Chinese herbal stores to buy medications in hope that the lump would become smaller."

“(would not go for screening programmes even if informed), because of fear”

"(heard) that (someone's) cancer was cured with some traditional medicine”

“My GP never encouraged me to go for (screenings) beforehand, so I never started (until after the usual time frame)"

Patient 5 (MALAY)

“Then after that I looked through the internet, then the symptoms in terms of cancer, or that. I didn’t find it that my lump can become cancerous”

"I mean they will be pressing the breast, or whatever. That’s why I thought it’s painful, or what, that’s why I didn't go for it.”

“also there is some of the, they write there that they will be pressing the thing, then the breast, I mean the cancer cell will go, travel all... "

“They say after chemo they will, I think the side effects after that, like nauseous, then feel hot, then dry mouth, all that. I can easily got an ulcer. That’s why I'm quite afraid of that also”

"(I use supplements) that I happen to see on Face Book, and Instagram”

Yes, (I thought that the supplements will have to make the breast lump) go away "

“I'm afraid to see the doctor. Because I'm afraid if they say I've got cancer”

Patient 6 (MALAY)

“because of the mind-set that I am healthy , it cannot be (the lump) is (cancerous).”

“when I go for mammogram, all that, I thought it’s very painful”

"Maybe scared of doctors, scared of all the operating, all the knives, all that that is shown on TV. That is the reason for why I don’t go to see a doctor.”

“If possible, I don’t want to go for chemo"

"You don’t want medicine? I said no. I find my own (medicine)”

“We tried the Malay traditional ones"

“Because I (was) really (scared of) disturb(ing)  everyone, so I just tried to ignore”

Patient 7 (CHINESE)

“(My friend) say mammogram is very, what, pain”

“my friends tell (me) the effects (of chemotherapy treatment)/(it) can make your hair like, drop like rain”

"Then I put medicine  and it's okay. Then after that it becomes spread”

“I use my (own medication) to cure the (disease)”

“I used Daktarin (before coming to the hospital)"

Patient 8 (CHINESE)

“(I didnt go to the doctor)Because I don’t feel pain”

“Afraid of the pain of mammogram”

“but I did take some hormones or whatnot, and then the lump also shrink.:

“Scared (of going to the doctor). Because sometimes we get negative answers or what”

“Sometimes it’s when you see the male doctor also, if he is male doctor also. That is also one that block me (from opening up)”

Patient 9 (CHINESE)

“I did go to Google search everything about my cough, breast cancer, about how to heal, how to cure the breast cancer alternatively.”

Patient 10 (MALAY)

“(Initially) I felt the lump but when I started to feel pain, then I go to check-up.”

“When you say mammogram, you know it must be very painful”

“Because there’s from US, Chris Beat Cancer, something like that, all these cancer institutes that are 100% cancer free/ they actually changed their diet. So initially before I did my chemo, I changed already my diet.”

Patient 11 (MALAY)

“Before the size growth (of the breast) there was actually a lump / So (only) when (my breast grew) bigger on one side, then (I decided to go to doctor)”

“so (the mammogram) is not really a fool-proof kind of detection/it's not 100%”

"I don’t have the confidence for whatever is recommended by the Western medicine”

“(I am most scared of) the side effect and the uncertainty (of the chemotherapy)”

“I do try like B17, beta glucan, there’s some more supplements which were recommended. Then after six months, it’s like not showing the result then I try starting on the Malay herbs.”

“As long as (the doctor) is female, it’s okay”

Patient 12 (MALAY)

“Daughter encouraged me to do the scan but I was too afraid:

“Only felt (that) radiotherapy was bad but chemotherapy (is) ok/I researched on Google (and) cancer.net”

Patient 13 (MALAY)

“I was afraid of the need for operations or procedures that I would need to go through”

“I delayed the visit to the doctor because I was scared and was trying to deny the visit to the doctor

Patient 14 (CHINESE)

"I thought it’s just a normal small lump … and I tend to be quite busy with work"

“It’s just I feel like I don’t  want to add my burdens to other people”

“Because if you personally see a person that is close to you and she has gone through all the treatments and she is suffering like that, you will tell yourself, I don’t want to go through all this.”

“Because maybe he’s a male doctor. I feel a bit shy to ask certain questions.”

“Then who is going to pay for you if you are going to stay?”

Patient 15 (MALAY)

“I just ignore, ignore it … until suddenly they get bigger and bigger”

“I don't like to bother my family”

“I am the sole breadwinner of the family”

Patient 16 (CHINESE)

"Admitted to the hospital due to pain "

"very very pain so admit hospital""

“busy with work and cannot make time to come down”

“noticed 2 years ago. Starting growing bigger and bigger with intermittent pain."

“Defaulted on polyclinic referral. As she was afraid of it being cancer.”

"I did not want to worry my parents"

"Read some information about breast cancer and her breast lump did not match the symptoms of the breast cancer/around the area with the lump… abnormal black mould"

Patient 18 (CHINESE) – translated from Chinese interview

“There was no pain so I ignored it”

“Avoids doctor. Do not want to take medication.”

“not aware of the symptoms of breast cancer”

Patient 19 (CHINESE)

“I used to go for MMG but the pain (deterred) her”

“I do not like others to worry for me.”

“Wants to bear the burden (herself).”

Patient 20 (MALAY)

“thought it was a normal lump…until it started growing""

"heard from someone about the radio/chemo side-effects due to social circle… all burnt after the radiotherapy"

"Didn’t know there will be a lump (in breast cancer)."

Patient 21 (MALAY)

“because the lump is very small , it’s under the nipple area, so it’s not on top”

“Yes, because I have one friend, she did her radiotherapy. The breast cancer that she had, the lump is near to the heart , so now, after the treatment, she got very easily tired, you see. So, she learned about it, and so she talked to us.”

Patient 22 (MALAY)

“Felt a lump in her breast”

“used a hot towel to compress the lump”

“When (she) found out about (her) lump, husband was sick.”

Patient 23 (MALAY)

“I was scared of the result, and I was scared I would get it.”

“I have never searched about breast cancer/I don't know what type of sickness it is”

“I prefer a female doctor.”
